# Postprandial Effect of Yogurt Enriched with Anthocyanins from Riceberry Rice on Glycemic Response and Antioxidant Capacity in Healthy Adults

**DOI:** 10.3390/nu12102930

**Published:** 2020-09-24

**Authors:** Tanisa Anuyahong, Charoonsri Chusak, Thavaree Thilavech, Sirichai Adisakwattana

**Affiliations:** 1 Phytochemical and Functional Food Research Unit for Clinical Nutrition, Department of Nutrition and Dietetics, Faculty of Allied Health Science, Chulalongkorn University, Bangkok 10330, Thailand; Tanisa.anuyahong@gmail.com (T.A.); charoonsri.c@gmail.com (C.C.); 2 Department of Food Chemistry, Faculty of Pharmacy, Mahidol University, Bangkok 10400, Thailand; Thavaree.thi@mahidol.ac.th

**Keywords:** yogurt, riceberry rice, postprandial, glycemia, antioxidant, anthocyanin

## Abstract

The pigment of riceberry rice has been reported to contain anthocyanins which act as a free radical scavenger and inhibitor of carbohydrate digestive enzymes. Since the probiotic yogurt incorporated with the pigment of riceberry rice extract was previously developed, the present study was aimed to investigate the acute effect of riceberry rice yogurt consumption on postprandial glycemic response, antioxidant capacity, and subjective ratings in healthy adults. In a cross-over design, 19 healthy participants were randomized to consume 350 g of yogurt supplemented with 0.25% *(w/w)* riceberry rice extract or the control yogurt. Postprandial plasma glucose, antioxidant status, and subjective ratings were measured at fasting and intervals (0–3 h) after ingestion of yogurt. The primary outcome was glycemic response; the secondary outcomes were plasma antioxidant capacity. In comparison to the yogurt control, riceberry rice yogurt reduced plasma glucose concentration after 30 min of consumption. The incremental area under the curve (iAUC) was significantly lower after riceberry rice yogurt load than after the control yogurt load. The consumption of riceberry yogurt caused an acute increase in plasma ferric reducing ability of plasma (FRAP), Trolox equivalent antioxidant capacity (TEAC), and oxygen radical absorbance capacity (ORAC) from the baseline values after 60 min of 0.25 ± 0.06 mM FeSO_4_, 253.7 ± 35.5 mM Trolox equivalents, and 166.8 ± 28.9 mM Trolox equivalents, respectively. Furthermore, the iAUCs for FRAP, TEAC, ORAC, and protein thiol were higher in riceberry yogurt consumption compared with the control yogurt (1.6-, 1.6-, 2.9-, and 1.9-fold, respectively). A decrease in iAUC for plasma malondialdehyde (MDA) concentration was also observed in the riceberry yogurt group. However, consumption of riceberry rice yogurt and control yogurt showed similar subjective rating scores of hunger, desire to eat, fullness, and satiety. In conclusion, acute consumption of riceberry rice yogurt suppressed postprandial glucose level and improved plasma antioxidant capacity in healthy volunteers.

## 1. Introduction

Yogurt, a semisolid fermented milk produced by lactic acid bacteria, is considered as an important functional food because of high nutritional content such as protein, calcium, vitamin B, phosphorus, magnesium, and potassium [1]. Recent studies reveal that consumption of yogurt fermented by lactic acid bacteria improves gastrointestinal health mediated through gut microflora, bowel transit, and immune response [2]. Especially, probiotic bacteria in yogurt have beneficial effects on host’s health by conferring protection against pathogenic bacteria and the prevention of gastrointestinal disorder such as irritable bowel syndrome (IBS), acute infectious diarrhea, or food intolerance [3,4]. In long-term intervention studies, the consumption of yogurt has been demonstrated to reduce the risk of developing metabolic diseases such as type 2 diabetes and cardiovascular diseases [5,6]. Interestingly, an intake of probiotic yogurt markedly improved glycemic control and increased antioxidant status in patient with type 2 diabetes [7].

In recent years, the role of certain plant-based foods on health benefit has drawn attention to consumers. Following this trend, several attempts have been made to the manufacture of yogurt containing the natural extract from fruits and vegetables in order to improve its nutritional, biological, and sensory properties [8]. For example, addition of cherries, berries, and grapes to yogurts increased the content of bioactive compounds such as phenolic acids and polyphenols [8,9]. Moreover, yogurt fortified with anthocyanins from chokeberries increased antioxidant activity and improved acceptable sensory properties [10]. Interestingly, combination of yogurt and plant-based foods also enhanced proliferation and survival of probiotics and modulated human microbiome [11,12].

Riceberry rice (*Oryza sativa* L.), a grain covered with deep-purple pigment, contains many nutrient components and bioactive compounds including anthocyanins, mainly cyanidin-3-glucoside (C3G) and peonidin-3-glucoside (P3G) [13]. It has been shown that the extract from riceberry rice possesses various biological activities such as antioxidant, anti-hyperglycemic, anti-hyperlipidemic, anti-glycation, and anti-inflammatory activities [13,14]. Consumption of riceberry rice bread had lower glycemic response with increased antioxidant capacity in healthy subjects when compared to Hom mali rice bread [15]. The probiotic yogurt enriched with anthocyanin-rich extract from riceberry rice was successfully developed and improved its functionality by increasing the amount of total phenolic compounds and antioxidant activity [16]. However, there is a substantial lack of evidence on postprandial glycemic response and antioxidant capacity of riceberry rice yogurt consumption in humans. Therefore, this study was aimed to investigated whether the consumption of riceberry rice yogurt affect the level of postprandial plasma glucose in healthy volunteers. The study was also investigated the effect of riceberry rice yogurt on plasma antioxidant capacity, the marker of lipid peroxidation and subjective appetite sensations.

## 2. Materials and Methods

### 2.1. Ethical Approval

The study was approved by the office of Ethics Review Committee for Research Involving Human Research Subjects, Human Science Group, Chulalongkorn University (COA No. 241/2018). The study began in January 2019 and completed in March 2019. The trial was registered at the Thai Clinical Trials Registry (study ID: TCTR20190118006). All subjects gave their written informed consent to participate. All information of participants was kept confidential. There were no major changes in the study protocol after initiation of the study.

### 2.2. Riceberry Rice Yogurt

The set-type yogurt was obtained from the report of Anuyahong et al. [16]. The ingredients consisted of whole milk, skimmed milk powder (3% *w/w*), and sucrose (5% *w/w*), with or without the anthocyanin-rich extract of riceberry rice (0.25% *w/w*). The yogurt contained *Streptococcus thermophilus*, *Lactobacillus delbrueckii* subsp. *bulgaricus*, *Lactobacillus acidophilus* LA-5 and *Bifidobacterium animalis* subsp. *lactis* BB-12. The proximate analysis of yogurt was performed by The Food Research and Testing Laboratory, Faculty of Science, Chulalongkorn University. The nutritional and phytochemical composition are reported in Table 1. 

### 2.3. Participants

Twenty-three subjects were recruited to participate in this study. The inclusion questionnaire was used to evaluate the eligibility criteria of participants. The inclusion criteria were as follow: (1) age of 18–40 years; (2) body mass index (BMI) of 18.5–22.9 kg/m^2^; (3) fasting blood glucose level < 100 mg/dL; (4) total cholesterol level < 200 mg/dL; (5) triglyceride level < 150 mg/dL; and (6) physically active. They were excluded if they met any of the following criteria: (1) pregnant or lactating; (2) presence of diabetes mellitus or insulin resistance; (3) use of dietary supplement or medication know to interfere with glucose homeostasis; (4) self-report of alcohol or tobacco products; and (5) allergy or intolerance to yogurt or dairy products. From this group, 23 subjects met the inclusion criteria.

### 2.4. Study Design

The study was a randomized-crossover trial with a one-week washout period. The subjects were randomly assigned to the ingestion of yogurt supplemented with or without 0.25% riceberry rice extract according to a sequence of random numbers, obtained from the online random number generator (www.randomizer.org). The process of randomization was performed by a principle researcher with a concealed allocation design. The participants were instructed not to take foods high in phytochemicals at least three days before each study period. Additionally, they were also asked to maintain stable habitual dietary intake and activity throughout their participation in the study period and to refrain from alcohol intake and heavy exercise for 24 h before each test. The primary outcome was glycemic response (postprandial glucose). The secondary outcomes were plasma antioxidant capacity (the ferric reducing ability of plasma, the Trolox equivalent antioxidant capacity, the oxygen radical absorbance capacity, the thiol group, and plasma malondialdehyde).

On the day of testing, the participants arrived at the Department of Nutrition and Dietetics, Faculty of Allied Health Sciences, Chulalongkorn University after overnight fast. Anthropometrics (body weight and height) were measured upon arrival at each session. Following baseline measurements, the fasting blood sample was collected from forearm vein with intravenous catheter by a registered nurse. Thereafter, the subjects were asked to the rate of hunger, fullness, desire to eat, and satiety with a visual analog scale (VAS) rating using a 10-cm scale from 0 (“not at all”) to 10 (“extremely”) at 0 min (before). They completely consumed 350 g of the study yogurt within a 10-min period. Blood samples were drawn at 15, 30, 60, 90, 120, 150, and 180 min, while subjective ratings were taken at 30, 60, 90, 120, 150, and 180 min postprandially. The subjects were restricted to drink water (<500 mL) during the study.

### 2.5. Biochemical Analysis

The blood samples were collected into blood colleting tube containing sodium fluoride and EDTA as anticoagulants for the measurement of glucose and antioxidant capacity, respectively. The plasma samples were separated by centrifuged at 3000 rpm for 15 min at 4 °C and kept at −80 °C for further analysis. The level of plasma glucose concentration was measured by glucose oxidase assay according to the manufacturer’s protocol (Glucose Liquicolor, HUMAN GmbH, Wiesbaden, Germany).

The ferric reducing ability of plasma (FRAP) assay in a redox-linked colorimetric reaction was performed according to a previous published report [17]. In brief, the plasma sample (10 µL) was mixed with 90 µL of freshly prepared FRAP reagent (0.3 M sodium acetate buffer (pH 3.6), 10 mM 2,4,6-Tri(2-pyridyl)-s-triazine (TPTZ) in 40 mM HCl, and 20 mM FeCl_3_). After incubation for 30 min at room temperature, the absorbance was read at 595 nm. The results were expressed as the EC (Equivalence concentration) value obtained from a standard curve of FeSO_4_.

The Trolox equivalent antioxidant capacity (TEAC) assay was measured using 2,2′-azinobis (3-ethyl benzothiazoline-6-sulfonic acid) (ABTS) radical (ABTS^●+^) [15], which was prepared by mixing 7 mM ABTS in 0.1 M PBS (pH 7.4) together with 2.45 mM K_2_S_2_O_8_ in distilled water (1:1, *v/v)*. After incubation for 16 h at room temperature, the ABTS^●+^ solution was diluted with 0.1 M PBS (pH 7.4) to adjust the absorbance between 0.9 and 1.0 at 734 nm. The plasma (10 µL) was incubated with the adjusted ABTS^●+^ solution (90 µL) for 6 min at the room temperature. The absorbance was recorded at 734 nm. The result of plasma TEAC was expressed as mM Trolox equivalents.

The oxygen radical absorbance capacity (ORAC) assay was performed according to a previous report [18]. Briefly, the 10× dilution of plasma in 0.1 M phosphate buffer saline (PBS), pH 7.4 (25 µL) was incubated with 4.8 nM sodium fluorescein in 75 mM PBS (150 µL) at 37 °C. After 10 min of incubation, 64 mM AAPH (25 µL) was added to the mixture. The fluorescence intensity was measured for 1 h with 2-min interval at excitation 485 nm and emission 535 nm. The ORAC value was calculated from the area under the curve (AUC) and expressed as µmol Trolox equivalents.

The thiol group level in plasma was measured using an Ellman’s assay [19] with slight modification. In brief, plasma sample (90 μL) was mixed with 130 μL of 2.5 mM of 5,5′-dithiobis-(2-nitrobenzoic acid) (DTNB) in 0.1 M PBS, pH 7.4 and incubated for 15 min at the room temperature. Then, the absorbance was measured at 410 nm. The plasma thiol level was calculated and expressed as μM L-cysteine equivalent.

Plasma malondialdehyde (MDA), a lipid peroxidation product, was quantified using thiobarbituric acid reactive substances (TBARS) assay. The plasma sample (200 μL) was mixed with trichloroacetic acid (10% *w/v*) and 50 mM 2,6-Di-tert-bytyl-4-methylphenol (BHT) and centrifuged at 12,000 rpm for 10 min [15]. The supernatant (200 μL) was separated and further mixed with 0.67% TBA before boiling at 100 °C for 10 min. After cooling down to room temperature, the absorbance of pink-colored of reaction was measured at 532 nm. Plasma MDA concentration was calculated from the calibration curve of MDA and expressed as µmol/L MDA.

### 2.6. Sample Size

The sample size was calculated following a previous study of postprandial response that reported a significant change in AUC for plasma glucose with a study power of 80% and alpha of 0.05 [20]. The sample size of 17 subjects were calculated with a confidence level of 95% (α = 5%) and power of 80%. Considering the 30% dropout, the final sample size was increased to 23 subjects.

### 2.7. Statistical Analysis

The results are expressed as mean ± SEM. The normality and homogeneity of the data was tested by Shapiro–Wilk test. Repeated-measures ANOVA was performed to determine the effect of treatment, time, and interaction of treatment and time followed by a paired *t* test to find the significance for each time point at *p* < 0.05. The incremental area under the curve (iAUC) for postprandial glucose, antioxidant status and lipid peroxidation (MDA) was calculated by using the trapezoidal rule integrated count areas above and below the fasting baseline concentration. Paired samples t-tests confirmed significant differences in the results from iAUC (*p* < 0.05).

## 3. Results

### 3.1. Participants

**Figure 1:**
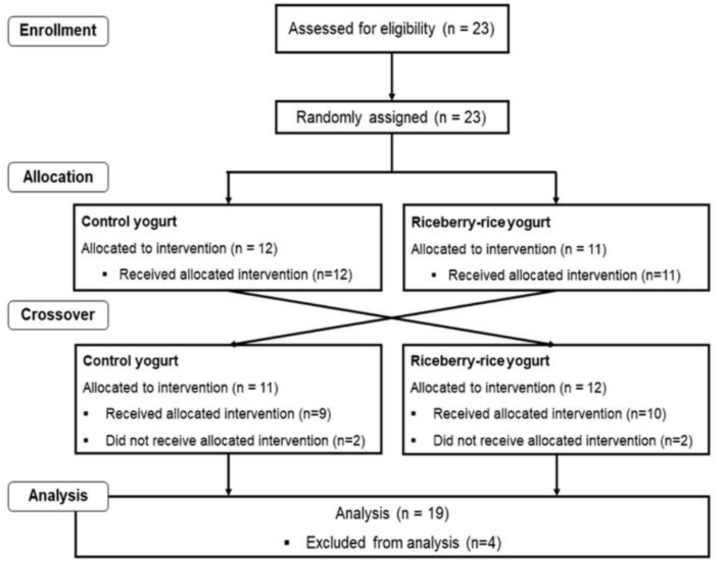
Consolidating Standards of Reporting (CONSORT) flow diagram of selection of study participants.

Twenty-three participants were recruited at the beginning and only nineteen participants (8 male and 11 female) completed the study. Four participants who did not receive intervention were excluded from analysis. The recruitment and enrollment data are presented in Figure 1. The characteristics of participants are reported in Table 2.

### 3.2. Postprandial Plasma Glucose

**Figure 2:**
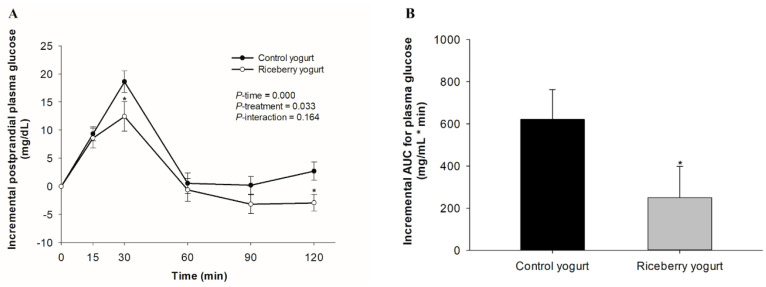
Incremental changes in postprandial plasma glucose concentration (**A**) and the incremental area under the curve (iAUC) for postprandial plasma glucose (**B**) in healthy participants after consuming either the control yogurt (●) or riceberry rice yogurt (○). Data are presented as mean ± SEM, *n* = 19. * *p* < 0.05 compared to the control yogurt.

Incremental changes in postprandial plasma glucose concentration after consumption of yogurts are demonstrated in Figure 2A. The peak of plasma glucose level was at 15 and 30 min after consumption of all yogurts. Postprandial plasma glucose concentration following riceberry yogurt was significantly lower than following the control at 30 and 120 min. As shown in Figure 2B, a reduction in iAUC of glucose for riceberry rice yogurt relative to the control was observed.

### 3.3. Postprandial Plasma Antioxidant Status

**Figure 3:**
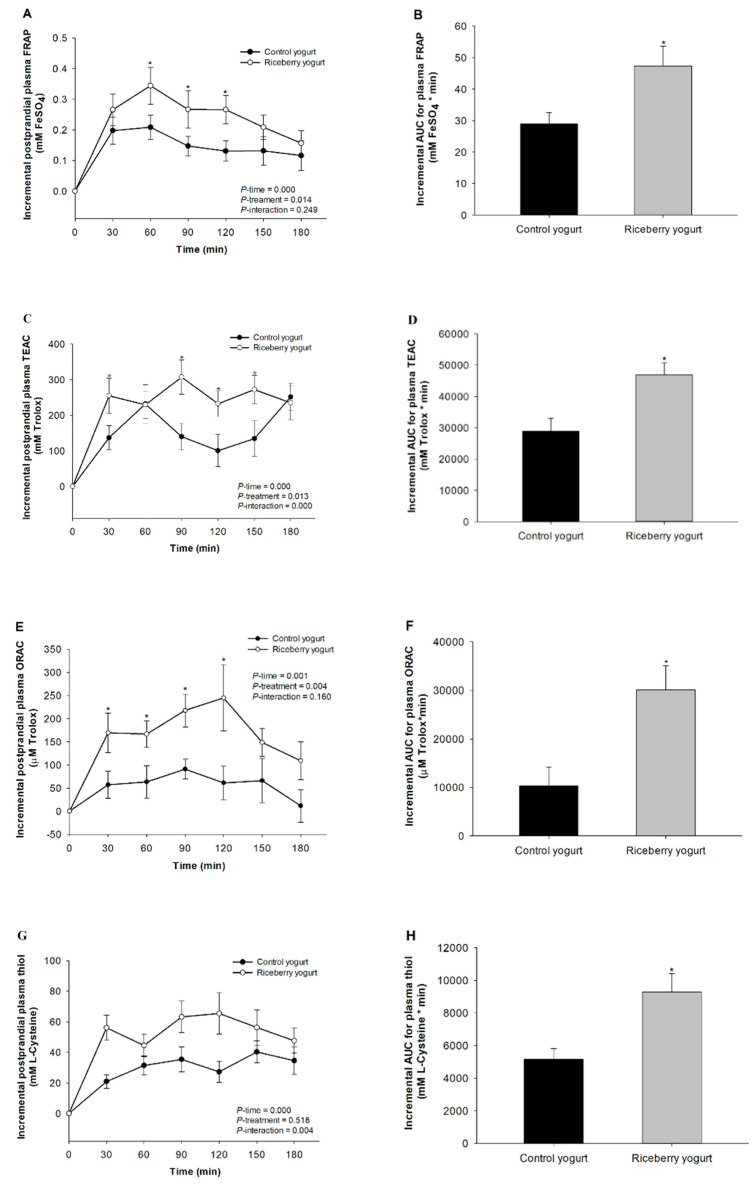

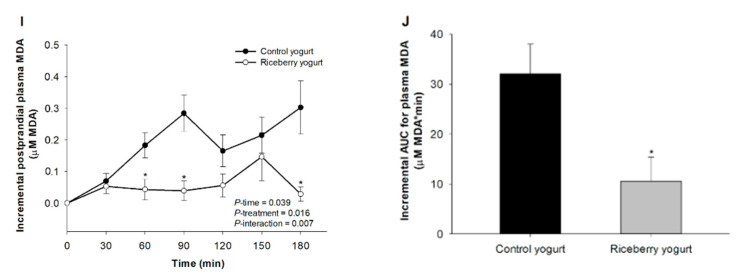
Incremental changes in postprandial plasma: ferric reducing ability of plasma (FRAP) (**A**); Trolox equivalent antioxidant capacity (TEAC) (**C**); oxygen radical absorbance capacity (ORAC) (**E**); thiol (**G**); and malondialdehyde concentration (MDA) (**I**). The incremental area under the curve (iAUC) for postprandial plasma: FRAP (**B**); TEAC (**D**); ORAC (**F**); thiol (**H**); and MDA (**J**) in healthy participants after consuming either the control yogurt (●) or riceberry rice yogurt (○). Data are presented as mean ± SEM, *n* = 19. * *p* < 0.05 compared to the control yogurt.

Compared with the control, the incremental changes in postprandial FRAP was significantly increased at 60, 90, and 120 min after consumption of riceberry rice yogurt (Figure 3A). The results show that consumption of riceberry rice yogurt resulted 1.6-fold greater in the iAUC of plasma FRAP level when compared with the control (Figure 3B).

Consumption of all yogurts caused a significant increase in TEAC above baseline for all time points. Plasma TEAC level at 30, 90, 120, and 150 min was significantly higher in participants who received riceberry rice yogurt than in those who consumed the control yogurt (Figure 3C). The iAUC of postprandial plasma TEAC was 1.6-fold greater in riceberry rice yogurt than in the control (Figure 3D).

Incremental changes in postprandial plasma ORAC after consumption of yogurts are presented in Figure 3E. The postprandial plasma ORAC level appeared immediately following intake of all yogurts and returned to the baseline level at 180 min. The plasma ORAC level was significantly higher for riceberry rice yogurt than the control at 30, 60, 90, and 120 min. In particular, the 2.9-fold increase in iAUC of plasma ORAC was observed for the subjects who consumed riceberry rice yogurt (Figure 3F).

Figure 3G presents incremental changes in postprandial plasma thiol after ingestion of yogurts. The postprandial plasma thiol level was elevated after consumption of all yogurts when compared to the baseline level. The results show that the postprandial plasma thiol level did not differ between riceberry rice yogurt and the control at each time point. Nevertheless, riceberry rice yogurt caused a 1.8-fold increase iAUC of plasma thiol, in comparison to the control yogurt (Figure 3H).

Incremental changes in postprandial plasma MDA concentration after consumption of yogurts are shown in Figure 3I. Plasma MDA concentration increased significantly from baseline following the control yogurt. Interestingly, consumption of riceberry rice yogurt markedly reduced the rise in plasma MDA concentration at 60, 90, and 180 min. A reduction in iAUC of postprandial plasma MDA (33%) was perceived following consumption of riceberry rice yogurt (Figure 3J).

### 3.4. Subjective Rating

**Figure 4:**
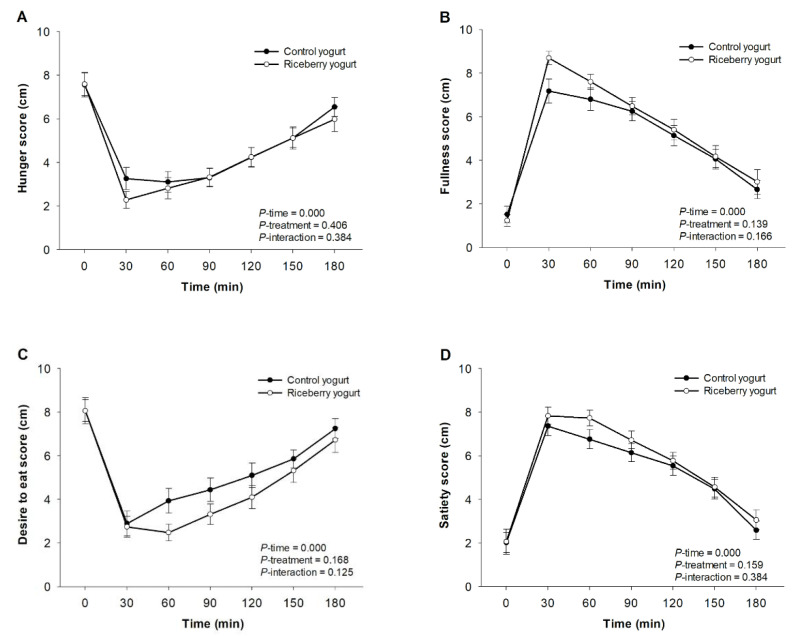
Incremental changes in subjective ratings for: hunger (**A**); fullness (**B**); desire to eat (**C**); and satiety (**D**) in healthy participants after consuming either the control yogurt (●) or riceberry rice yogurt (○). Data are presented as mean ± SEM, *n* = 19.

The subjective rating scores of hunger, fullness, desire to eat, and satiety after consumption of yogurts are illustrated in Figure 4A–D. All yogurts markedly reduced the score of hunger and desire to eat and increased the score of satiety and fullness after 30 min of ingestion as compared to baseline. However, there were no statistically significant differences in the rating score of hunger, fullness, desire to eat, and satiety among all yogurts.

## 4. Discussion

Yogurt has received considerable attention as a potential approach to reduce the risks of weight gain, obesity, type 2 diabetes, and cardiovascular diseases [5,6]. Especially, several studies have reported the successful fortification of yogurt with bioactive compounds from edible plants such as green tea, black tea, white tea [21], chamomile [22], strawberry pulp [23], and aronia juice [10]. In our previous study, the supplementation of probiotic yogurt with anthocyanins from riceberry rice provided bioactive compounds and increased its functionality by increasing total phenolic content (TPC), cyanidin-3-glucoside (C3G), and peonidin-3-glucoside (P3G) concomitant with the elevation of DPPH radical scavenging activity and ferric reducing antioxidant power. In gastrointestinal digestion, this yogurt produced higher release of TPC and FRAP than the control [16]. Therefore, this was the first human study to investigate whether riceberry rice yogurt decreases postprandial glycemic response and improves antioxidant capacity in healthy volunteers. A reduction in the postprandial glucose excursion (40.23%) after consumption of riceberry rice yogurt was observed in healthy subjects. Cross-over studies have explored acute effects riceberry rice bread (50 g) on postprandial glycemic and insulin response in healthy volunteers [15]. After the riceberry rice bread intake, the AUC was 60% lower in comparison to jasmine rice bread. Furthermore, it was found to produce an attenuated postprandial insulin concentration after 15 min of consumption. The main reason to explain these effects is the ability of riceberry rice and its phytochemical compounds to inhibit carbohydrate digestive enzymes [15,24,25]. A previous study supports this reason that the anthocyanin-rich extract of riceberry rice was capable of inhibiting intestinal α-glucosidase such as maltase and sucrase [14]. Especially, C3G and P3G, the major anthocyanins identified in riceberry rice extract, was proved to be effective pancreatic α-amylase and α-glucosidase inhibitors [14,26,27]. Since lactose from milk was the major source of carbohydrate in the yogurt, a further study is needed to investigate the inhibitory effect of anthocyanins on lactase activity.

Postmeal hyperglycemia and glycemic fluctuations induces excessive production of reactive oxygen species (ROS). The excessive formation of ROS may be a contributing factor for induction of pathological changes related to the development of cardiovascular diseases [28]. Interestingly, dietary antioxidants help scavenge and neutralize excessive and inappropriate ROS, sequencing to balance against oxidant condition [29]. Scientific evidence suggests that consumption of phytochemical-rich plants improved plasma antioxidant capacity and reduced lipid peroxidation in humans [30,31,32]. The different assays have been commonly used to measure plasma antioxidant capacity such as ORAC, TEAC, and FRAP. The ORAC assay refers the ability of antioxidant molecules to inhibit peroxyl radical induced oxidation [33], whereas the TEAC assay indicates the ability of hydrogen-donating antioxidants to neutralize a radical cation in both lipophilic and hydrophilic environments [34]. FRAP assay could reflect the ability of antioxidants to reduce the reaction of Fe3+/Fe2+ couple. In addition, protein thiols in plasma has multifaceted functions, including a pivotal role in antioxidant defense [35]. Compared to fasting state, consumption of yogurt control slightly increased plasma antioxidant capacity (FRAP, TEAC, and ORAC) and protein thiol level. This result is entirely due to yogurt containing milk proteins and natural substances which have antioxidant activity [36]. Remarkably, plasma FRAP, TEAC, ORAC, and protein thiol levels were high 180 min after riceberry rice yogurt consumption, in comparison with the yogurt control. Increases in postprandial antioxidant capacity are supported by in vitro studies indicating that yogurt supplemented with riceberry rice extract (0.25%) had 4.8-fold higher FRAP than the control yogurt [16]. Similar results were also reported by Chusak et al. who found a greater increase in plasma FRAP following riceberry rice bread consumption [15]. A notable result from our study was a marked increase in plasma MDA above baseline after an intake of yogurt control, indicating that diet could induce postprandial lipid oxidation. This alteration was noticeably attenuated by riceberry rice yogurt consumption. These findings led us to hypothesize that riceberry rice yogurt would decrease postprandial oxidative stress related to their antioxidant activity.

Other clinical studies have also shown improvement in plasma antioxidant capacity together with reduction of lipid peroxidation following consumption of anthocyanin-rich plants such as butterfly pea flower [37], Chilean berry [38], and açai berry [31]. It is suggested that the improvement of plasma antioxidant status may be partly attributed to the antioxidant activity of phenolic compounds [37]. In this context, C3G and P3G, the incorporated active ingredients in riceberry rice yogurt, have been recognized as antioxidant agents as represented by FRAP, TEAC, and ORAC assays [39,40]. In addition, C3G and P3G had the ability to reduce the formation of lipid peroxidation in UVB irradiation model and vitamin E-depleted rat [41,42]. Interestingly, after consumption of anthocyanin-rich strawberries and chokeberries, mainly C3G reduced plasma MDA concentration by 31% and 46%, respectively [43,44]. Through these actions, C3G and P3G in riceberry rice yogurt may play a role in an increase in plasma antioxidant capacity, leading to decrease in lipid peroxidation. However, other phytochemical compounds in riceberry rice may influence postprandial antioxidant capacity. Therefore, the quantification of the postprandial concentration of individual polyphenol after riceberry yogurt consumption is needed, which may help explore the role of riceberry rice yogurt in suppressing postprandial oxidative stress.

Visual analog scales (VAS) are relievable tools for the evaluation of subjective appetite sensation about hunger, fullness, desire to eat, and satiety [45]. In our study, the scores of all parameters did not show any significant differences between riceberry rice yogurt and the control yogurt. This finding is in agreement with the earlier study that bread made from riceberry rice did not alter subjective rating scores of hunger, fullness, desire to eat, and satiety in healthy adults. In addition, no change in postprandial level of glucagon-like peptide-1 (GLP-1), an incretin hormone, was detected following consumption of riceberry rice bread [15]. GLP-1 is responsible for the stimulation of insulin secretion, inhibition of glucagon secretion and gastric emptying, and regulation of appetite and satiety [46]. Interestingly, anthocyanins stimulated the secretion of GLP-1 from Murine GLUTag cell line [47]. We hypothesized that consumption of riceberry rice yogurt could not modulate satiety and appetite through the stimulation of GLP-1, possibly as a result from a small amount of anthocyanin in yogurt and its low bioavailability. Furthermore, we acknowledge some potential limitations to the current study. First, we did not introduce a full meal to consume with riceberry yogurt. Other macro- and micronutrients may interfere the postprandial effect of riceberry rice yogurt on plasma glucose and antioxidant capacity. Moreover, this study was only a relatively young and healthy population; older age subjects were not included to increase the homogeneity of postprandial response.

## 5. Conclusions

The present findings indicate that consumption of riceberry rice yogurt had a favorable effect in reducing postprandial plasma glucose and plasma MDA with improvement of plasma antioxidant status. With respect to appetite ratings, no significant change in scores of fullness, desire to eat, and satiety was observed following consumption of riceberry yogurt. The results suggest that riceberry rice yogurt could be a healthy food for improving the postprandial glycemic and antioxidant response in humans. The further study should determine the long-term effect of riceberry rice yogurt consumption in other populations at risk for chronic diseases following meals.

## Figures and Tables

**Table 1:** The nutritional composition and phytochemical compounds of yogurt for one serving size (350 g).

Composition	Control Yogurt	Riceberry Rice Yogurt
Energy (kcal)	349.1	349.6
Carbohydrate (g)	43.5	43.9
Total dietary fiber	n.d	n.d
Protein (g)	13.8	13.8
Fat (g)	13.3	13.2
Moisture (g)	275.9	275.5
Ash (g)	3.5	3.6
Total polyphenol content Anthocyanins	16.1	28.1
Cyanidin-3-glucoside	n.d	17.4
Peonidin-3-glucoside	n.d	7.9

n.d = not detected.

**Table 2:** Baseline characteristics of participants.

Parameters	Mean ± SEM
Age (years)	28.1 ± 3.0
Height (m)	1.65 ± 0.1
Weight (kg	58.3 ± 2.2
BMI (kg/m^2^)	21.2 ± 0.4
Fasting plasma glucose (mg/dL)	83 ± 1.0
Total cholesterol (mg/dL)	167.3 ± 8.1
Serum triglyceride (mg/dL)	53.0 ± 3.8
LDL-C (mg/dL)	113.5 ± 6.6
HDL-C (mg/dL)	43.2 ± 3.5
Creatinine (mg/dL)	0.7 ± 0.1
Blood urea nitrogen (BUN) (mg/dL)	11.6 ± 0.5

All values are mean ± SEM, *n* = 19.
